# Non‐viral Gene Therapy for Melanoma Using Lysenin from *Eisenia Foetida*

**DOI:** 10.1002/advs.202306076

**Published:** 2024-03-06

**Authors:** Min Ren, Ling Yang, Liming He, Jie Wang, Wei Zhao, Chunli Yang, Shuai Yang, Hao Cheng, Meijuan Huang, Maling Gou

**Affiliations:** ^1^ Department of Biotherapy Cancer Center and State Key Laboratory of Biotherapy West China Hospital Sichuan University Chengdu Sichuan 610041 China; ^2^ Huahang Microcreate Technology Co., Ltd Chengdu Sichuan 610041 China; ^3^ Division of Thoracic Tumor Multimodality Treatment and Department of Medical Oncology Cancer Center West China Hospital Sichuan University Chengdu Sichuan 610041 China

**Keywords:** cancer gene therapy, *Eisenia foetida*, Lysenin, nanomedicines, pore‐forming proteins

## Abstract

Earthworms, long utilized in traditional medicine, serve as a source of inspiration for modern therapeutics. Lysenin, a defensive factor in the coelom fluid of the earthworm *Eisenia fetida*, has multiple bioactivities. However, the inherent toxicity of Lysenin as a pore‐forming protein (PFP) restricts its application in therapy. Here, a gene therapy strategy based on Lysenin for cancer treatment is presented. The formulation consists of polymeric nanoparticles complexed with the plasmid encoding Lysenin. After transfection in vitro, melanoma cells can express Lysenin, resulting in necrosis, autophagy, and immunogenic cell death. The secretory signal peptide alters the intracellular distribution of the expressed product of *Lysenin*, thereby potentiating its anticancer efficacy. The intratumor injection of *Lysenin* gene formulation can efficiently kill the transfected melanoma cells and activate the antitumor immune response. Notably, no obvious systemic toxicity is observed during the treatment. Non‐viral gene therapy based on *Lysenin* derived from *Eisenia foetida* exhibits potential in cancer therapy, which can inspire future cancer therapeutics.

## Graphical abstract

A nanoparticle‐mediated gene therapy based on Lysenin, an exogenous pore‐forming protein from earthworm *Eisenia foetida*, holds promise for cancer treatment by directly killing the transfected cancer cells and inducing the anticancer immunity, which inspires the development of future cancer therapeutics related to pore‐forming proteins.

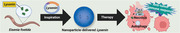

## 1 Introduction

Earthworms, classified within the Oligochaeta class of the phylum Annelida, are recognized to contain various biologically active substances.^[^ ^1^ ^]^ They have long been used as therapeutic preparations for various diseases. Lumbrokinase (LK) extracted from earthworms has been widely utilized clinically in China as an antithrombotic agent. LK capsules are also used as health supplements in many countries to support circulatory health. In addition, the antitumor activity of earthworms has received attention recently, inspiring several new cancer therapeutic strategies. Research has demonstrated that both earthworm extract (EE) and coelomic fluid (CF) have antitumor effects. For example, EE has shown significant capability to promote tumor apoptosis and reduce tumor size in vivo.^[^ ^2^ ^]^ CF can inhibit the proliferation of squamous cell carcinoma‐9 cell line and enhance the apoptosis of A549 human lung cancer cells.^[^ ^3^, ^4^ ^]^ Identifying specific active antitumor factors is beneficial for developing potential antitumor medicine.

Lysenin is a β‐pore‐forming toxin (β‐PFT) produced by the coelomocytes in the CF of earthworm *Eisenia foetida*.^[^ ^5^, ^6^ ^]^ Like most PFTs, Lysenin is secreted as an inactive, soluble monomer.^[^ ^7^ ^]^ These monomers strongly combine with sphingomyelin (SM) in the eukaryotic plasma membranes. They can form a nonameric transmembrane pore with a diameter of 3 nm on the target membranes and result in consequent cell lysis,^[^ ^8^, ^9^ ^]^ which drove us to explore the potential application of Lysenin in cancer treatment. However, vertebrate cells contain abundant SM in the outer leaflet of the plasma membrane, which makes Lysenin toxic to vertebrate spermatozoa and cultured mammalian cells.^[^ ^10^, ^11^, ^12^ ^]^ The severe hemolysis of Lysenin and its potential toxicity to normal cells have largely precluded its therapeutic applications. Therefore, we designed a gene formulation based on Lysenin to exploit its antitumor potential.

Gene delivery systems mainly include viral vector systems and non‐viral vehicle systems. Virus vectors have the characteristics of high delivery efficiency and broad host range. However, the limited capacity, easy clearance by the immune system, and potential bio‐risks have raised delivery and safety concerns.^[^ ^13^, ^14^, ^15^, ^16^, ^17^ ^]^ The non‐viral vehicles, majorly including lipoplexes and polyplexes, have the advantages of low cost, easy preparation on a large scale, good safety, and low immunogenicity, thus having garnered extensive attention recently.^[^ ^14^, ^18^, ^19^ ^]^ Due to the versatile physicochemical characteristics of polymers, polymeric vehicles have become promising tools for drug delivery.^[^ ^20^, ^21^, ^22^ ^]^ Several clinical trials of the polymeric nanoparticles for gene delivery have already been carried out.^[^ ^17^, ^23^ ^]^ Nanoparticle‐delivered gene therapy shows potential application in cancer therapy.

**Scheme 1:**
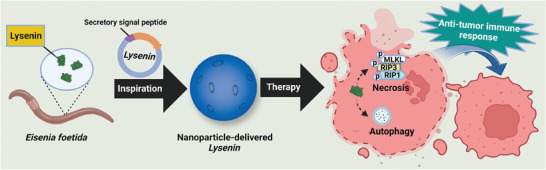
Non‐viral gene therapy for melanoma using Lysenin from the earthworm *Eisenia foetida* via directly killing the transfected cancer cells and inducing anticancer immunity.

Melanoma is a malignant tumor.^[^ ^24^, ^25^ ^]^ The FDA has approved the granulocyte‐macrophage colony‐stimulating factor engineered herpes simplex virus type I for the local treatment of malignant melanoma.^[^ ^26^ ^]^ FixVac, a nanoparticulate liposomal RNA vaccine, is under investigation in a dose‐escalation phase I trial for advanced melanoma.^[^ ^27^ ^]^ Nevertheless, the exploration for safer and more effective drugs remains ongoing. In this study, we used a biodegradable polymeric nanoparticle to mediate *Lysenin* for melanoma treatment. Our results indicated that the *Lysenin* gene formulation could efficiently suppress the tumor in vivo by directly killing tumor cells through the induction of necrosis and autophagy while also generating the antitumor immunity (as shown in **Scheme** **1**). Our work provided a promising non‐viral gene therapy strategy using Lysenin for melanoma treatment, which would inspire the development of future cancer therapeutics based on pore‐forming proteins (PFPs).

## 2 Results

### 2.1 The In Vitro Antitumor Activity of *Lysenin* Gene Formulation

**Figure 1:**
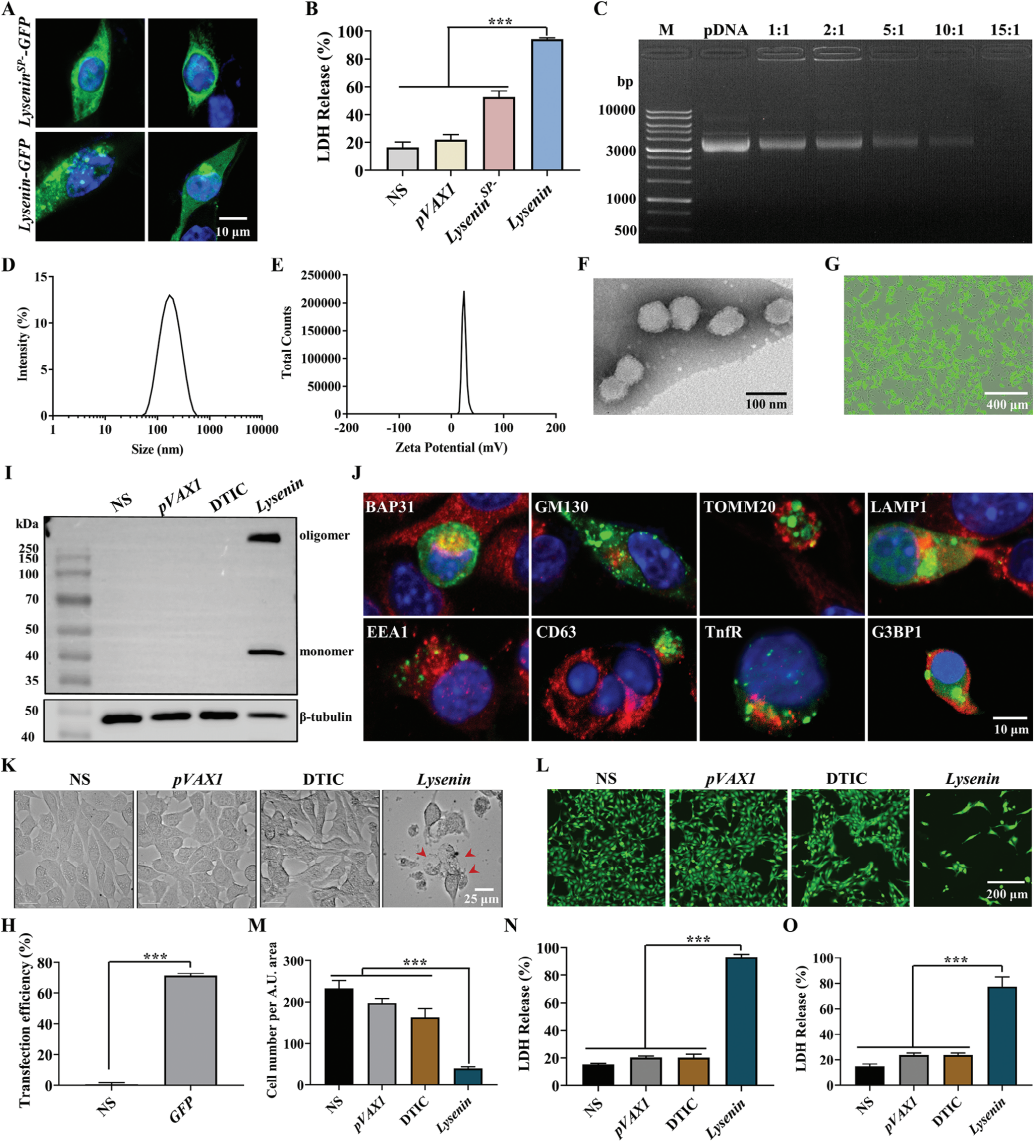
*Lysenin* gene formulation inhibited the growth of melanoma cells in vitro. A) The intracellular distribution of the expressed proteins of *Lysenin^SP−^‐GFP* and *Lysenin‐GFP*. Scale bar, 10 µm. B) The cytotoxicity of *Lysenin^SP−^* and *Lysenin* gene formulations to B16‐F10 melanoma cells (*n* = 3). ^***^ *p* < 0.001. C) The combining ability of nanoparticles and plasmids was evaluated by the agarose gel retardation assay. D,E) The hydrodynamic size and zeta potential of the *Lysenin* gene formulation. F) A representative TEM image of *Lysenin* gene formulation. Scale bar, 100 nm. G,H) The transfection efficiency of *GFP* nanocomplexes on B16‐F10 cells detected by flow cytometry (*n* = 3). Scale bar, 400 µm. ^***^ *p* < 0.001. I) Detection of the protein expression of *Lysenin* gene formulation using anti‐Flag antibody by WB. J) The relative location of the secretory signal peptide‐mediated Lysenin‐GFP to various cell components. Scale bar, 10 µm. K) The optical images of B16‐F10 melanoma cell of each group. Red arrows indicate balloon‐like cells. Scale bar, 25 µm. L,M) The detection of B16‐F10 melanoma cell viability and cell density by Calcein AM (*n* = 3). Scale bar, 200 µm. ^***^ *p* < 0.001. N) The detection of B16‐F10 murine melanoma cells’ LDH release (*n* = 3). ^***^ *p* < 0.001. O) The detection of A375 human melanoma cells’ LDH release (*n* = 3). ^***^ *p* < 0.001.

Inspired by the earthworm *Eisenia foetida* in defending invaders via Lysenin, we designed a new non‐viral gene formulation for melanoma treatment using Lysenin. The gene formulation consisted of two portions. One portion was the nanoparticle self‐assembled from monomethoxy poly (ethylene glycol)‐poly (d,l‐lactide) (mPEG‐PDLLA) and *N*‐[1‐(2,3‐dioleoyloxy) propyl]‐*N*,*N*,*N*‐trimethylammonium chloride (DOTAP), as synthesized according to our previous report.^[^ ^28^ ^]^ The other was the plasmid carrying the therapeutic gene. Given that some PFPs could form oligomeric pores on plasma or organelle membranes,^[^ ^29^, ^30^, ^31^, ^32^ ^]^ we designed two gene formulations based on Lysenin. One was without signal peptide (designated as *Lysenin^SP−^*), and the other was led by interleukin‐2 (IL‐2) secretory signal peptide (designated as *Lysenin*). In order to observe protein expression and distribution in tumor cells, we constructed the fusion expression vector of *Lysenin^SP−^* and enhanced green fluorescent protein (*Lysenin^SP−^‐GFP*) as well as *Lysenin‐GFP*. The fluorescence images showed that the expressed proteins of *Lysenin^SP−^‐GFP and Lysenin‐GFP* were distributed within the B16‐F10 melanoma cells with different patterns. The Lysenin^SP−^‐GFP was dispersedly distributed within the cell, while Lysenin‐GFP was more likely to form punctum‐like aggregates within the cells, indicating that the secretory signal peptide‐mediated Lysenin could not be successfully secreted out of the cells (**Figure** **1**A). Subsequently, we investigated the antitumor effects of two gene formulations on B16‐F10 melanoma cells. LDH release assay, often used as a cytotoxicity assessment, revealed that the *Lysenin* gene formulation had more excellent antitumor activity than the *Lysenin^SP^* ^‐^ gene formulation (Figure 1B). Therefore, we carried out the follow‐up study on *Lysenin* gene formulation.

First, we prepared and characterized the *Lysenin* gene formulation. The formulation was prepared using the previous method.^[^ ^28^ ^]^ The combining ability of nanoparticles and plasmids was evaluated by the agarose gel retardation assay. The results showed that the plasmids were completely encapsulated when the mass ratio of the nanoparticles to plasmids was 15:1 (Figure 1C). The hydrodynamic size and zeta potential of the gene formulations were 189 ± 16 nm and 24 ± 0.7 mV, respectively (Figure 1D,E). The images obtained via transmission electron microscopy (TEM) demonstrated that the gene formulations were spherical nanoparticles of 81 ± 9 nm in diameter (Figure 1F). The fluorescence detection of the reporter gene *GFP* revealed that the nanoparticle‐mediated gene delivery system could effectively transfect B16‐F10 melanoma cells with a 71.4% transfection rate (Figure 1G,H). Our findings suggested that the gene formulation had good characteristics and could be further studied for its antitumor effect.

Dacarbazine (DTIC), a chemotherapy drug in the clinical treatment of melanoma, was used as a positive control group. We also set a negative control group (NS) and an empty plasmid control group (*pVAX1*). Since the nucleic acid sequence of the Flag label was added behind the open reading frame of the recombinant *Lysenin* plasmid, we detected the protein expression of the *Lysenin* gene formulation using an anti‐Flag antibody by western blot (WB). Figure 1I showed that Lysenin protein was expressed as a monomer about 43 kDa and could form SDS‐resistant oligomers larger than 170 kDa, supported by the previous reports.^[^ ^6^, ^33^ ^]^ Furthermore, we explored the localization of the expressed protein in the cells transfected with *Lysenin‐GFP*. The marker proteins of membranous organelles were labeled with fluorescent‐conjugated antibodies. The endoplasmic reticulum, Golgi apparatus, mitochondria, lysosomes, early endosomes, late endosomes, and recycling endosomes were marked by hallmark proteins B‐cell receptor‐associated protein 31 (BAP31), Golgi matrix protein 130 (GM130), translocase of outer mitochondrial membrane 20 (TOMM20), lysosomal‐associated membrane protein 1 (LAMP1), early endosome antigen 1 (EEA1), cluster of differentiation 63 (CD63), and transferrin receptor (TfnR), respectively. The results showed that the expressed products of *Lysenin‐GFP* did not co‐localize with the marker proteins of these organelles (Figure 1J), indicating that some specific factors might affect the secretion process. Given that stress granules (SGs) are cytosolic biomolecular condensates that assemble in response to cellular stress,^[^ ^34^ ^]^ we also examined whether the signal peptide‐mediated Lysenin could form SGs. Through the immunofluorescence staining of G3BP stress granule assembly factor 1 (G3BP1), a key protein in the formation of SGs, we did not observe the SGs formation (Figure 1J), indicating that the location of the signal peptide‐mediated Lysenin remained to be revealed in future studies.

We further tested the killing effect of *Lysenin* gene preparation on B16‐F10 murine melanoma cells. We observed that many of the cells in the *Lysenin* group became round and floated in the medium after medication treatment for 24 h, and some cells were balloon‐like (Figure 1K). Intracellular esterase activity was often used to evaluate cell viability, which could be indicated by Calcein AM staining. The experimental results showed that the cell viability of the *Lysenin* group was significantly decreased compared with that of the NS group, *pVAX1* group, and DTIC group (Figure 1L,M). LDH release was an important indicator for assessing cytotoxicity and cell membrane integrity. Our results exhibited that *Lysenin* could strongly induce the LDH release of the B16‐F10 murine melanoma cells, reaching 93.2%. It indicated that the *Lysenin* gene formulation strongly damaged the plasma membrane and had a good killing effect on B16‐F10 murine melanoma cells. (Figure 1N). The same phenomenon was observed on the A375 human melanoma cells (Figure 1O). Taken together, these results demonstrated the anticancer activity of our gene formulation.

### 2.2 The Cell Death Mechanism Induced by *Lysenin* Gene Formulation

**Figure 2:**
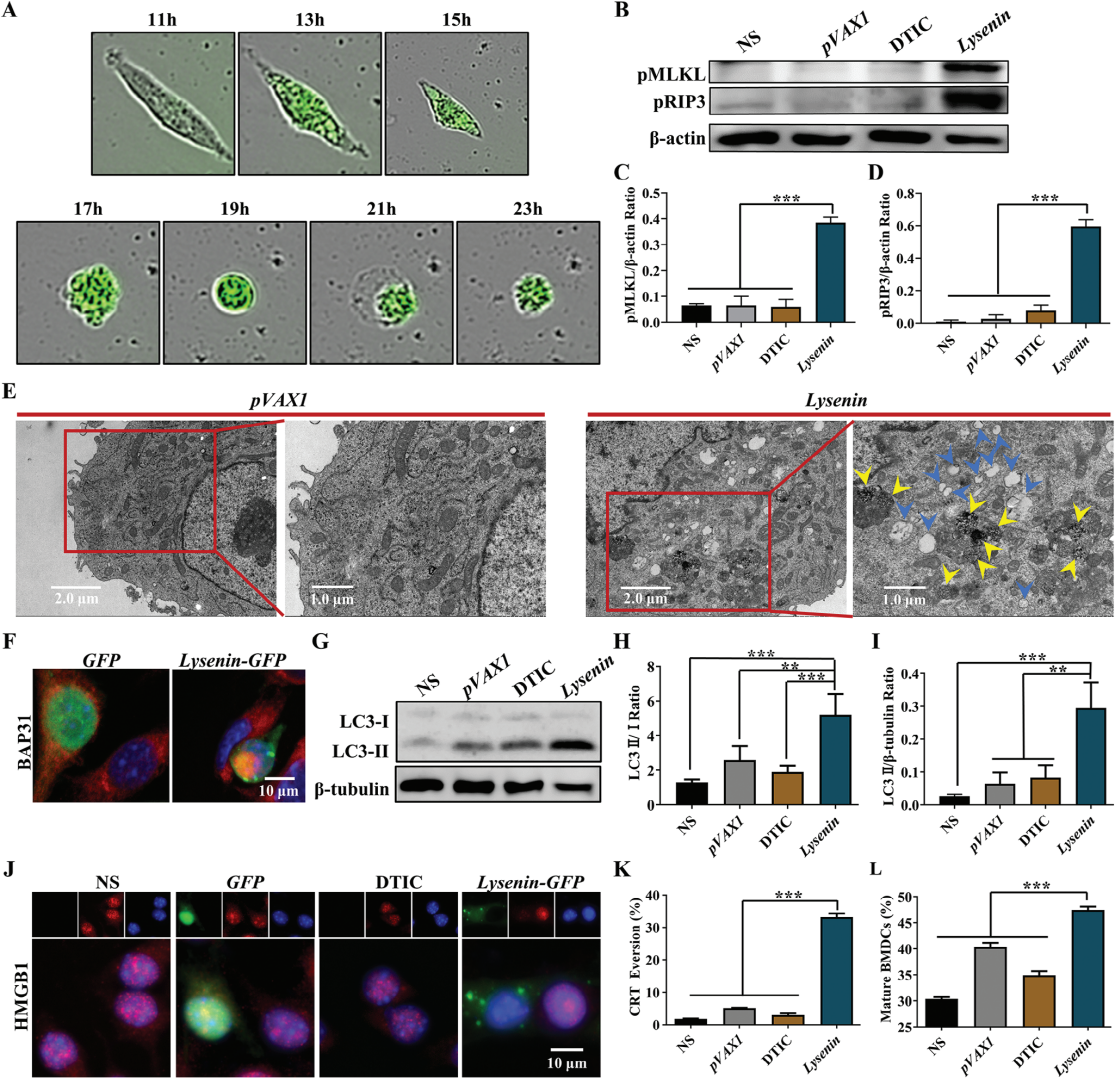
*Lysenin* gene formulation in killing tumor cells is associated with necrosis, autophagy, and immunogenic cell death. A) The morphological changes of B16‐F10 cells transfected with *Lysenin‐GFP*. B) The detection of necrosis‐related proteins pMLKL and pRIP3 in B16‐F10 cells treated with *Lysenin* gene formulation by WB. C,D) The statistical graphs of their ratios to the loading control β‐actin (*n* = 3). ^***^ *p* < 0.001. E) Representative TEM images showed the autophagosome and abnormal endoplasmic reticulum in B16‐F10 cells after *Lysenin* gene formulation treatment. NOTE: Yellow arrows indicate autophagosomes; blue arrows indicate abnormal endoplasmic reticulum. Scale bars, left panel, 2.0 µm; right panel, 1.0 µm. F) Immunofluorescent staining of the hallmark protein BAP31 of the endoplasmic reticulum. Scale bar, 10 µm. G) The effect of *Lysenin* gene formulation on the expression of LC3 in B16‐F10 cells detected by WB. H,I) The statistical graphs of LC3II/I ratio and LC3II/β‐tubulin (*n* = 3). ^**^ *p* < 0.01, ^***^ *p* < 0.001. J) *Lysenin* gene formulation reduced the HMGB1 protein content in the nucleus of B16‐F10 cells. Scale bar, 10 µm. K) Flow cytometry detection of CRT eversion in B16‐F10 cells induced by *Lysenin* gene formulation (*n* = 3). ^***^ *p* < 0.001. L) The ability of B16‐F10 cells treated with *Lysenin* gene formulation to promote BMDCs maturation was detected by flow cytometry (*n* = 3). ^***^ *p* < 0.001.

In order to observe the cell death induced by *Lysenin* gene formulation, we used a long‐term dynamic cell observation and analysis system to study the morphological changes of B16‐F10 melanoma cells transfected with *Lysenin‐GFP*. The moment of adding the transfection complexes into the culture was marked as 0 h. As the green fluorescence gradually increased within the cells, they became smaller and shrank into irregular shapes, reminiscent of the process of necrosis (**Figure** **2**A). The phosphorylation of mixed lineage kinase domain‐like protein (MLKL) and its upstream receptor‐interacting protein 3 (RIP3) were essential for the occurrence of necrosis.^[^ ^35^ ^]^ Our experiments showed that MLKL and RIP3 in the *Lysenin* group were phosphorylated (Figure 2B–D), demonstrating that *Lysenin* gene formulation indeed induced the necrosis of B16‐F10 cells.

Then, we performed ultrastructural studies using TEM, which could enable visual observation of the intracellular organelles. The TEM images revealed endoplasmic reticulum dilatation and obvious autophagosomes in B16‐F10 cells treated with *Lysenin* gene formulation, but no such phenomenon was observed in cells transfected with control plasmid *pVAX1* (Figure 2E). The immunofluorescence results of BAP31 also showed abnormalities in the endoplasmic reticulum of B16‐F10 cells treated by *Lysenin* gene formulation (Figure 2F). In order to verify the formation of autophagosomes, we detected the autophagic marker microtubule‐associated protein 1 light chain 3 (LC3) via WB assay. The results further demonstrated that *Lysenin* gene formulation induced autophagy (Figure 2G–I).

The release of damage‐related molecular patterns (DAMPs), which could induce an immune response in vivo, was detected. The efflux of high mobility group protein B1 (HMGB1) and calretinin (CRT) eversion were two important indicators to assess the release of DAMPs. Immunofluorescence analysis showed that the expression of HMGB1 in the nucleus was significantly decreased after *Lysenin* gene formulation treatment (Figure 2J), suggesting that HMGB1 protein might be expelled out of the cell. CRT eversion was detected by flow cytometry, and the statistics demonstrated that 33.2% of B16‐F10 cells in the *Lysenin* group came into being CRT eversion (Figure 2K). Dendritic cells (DCs) maturation was another important indicator for evaluating antitumor immune response in vivo and played a significant role in activating cytotoxic T cells. The co‐cultivation of murine bone marrow‐derived DCs (BMDCs) and B16‐F10 melanoma cells treated with *Lysenin* gene formulation significantly promoted the maturation of BMDCs in vitro (Figure 2L). Our results indicated that the *Lysenin* gene formulation could induce immunogenic death of B16‐F10 murine melanoma cells. In conclusion, the mechanisms of *Lysenin* gene formulation in killing tumor cells were associated with necrosis, autophagy, and immunogenic cell death.

### 2.3 The In Vivo Inhibition Effect of *Lysenin* Gene Formulation on B16‐F10 Melanoma

**Figure 3:**
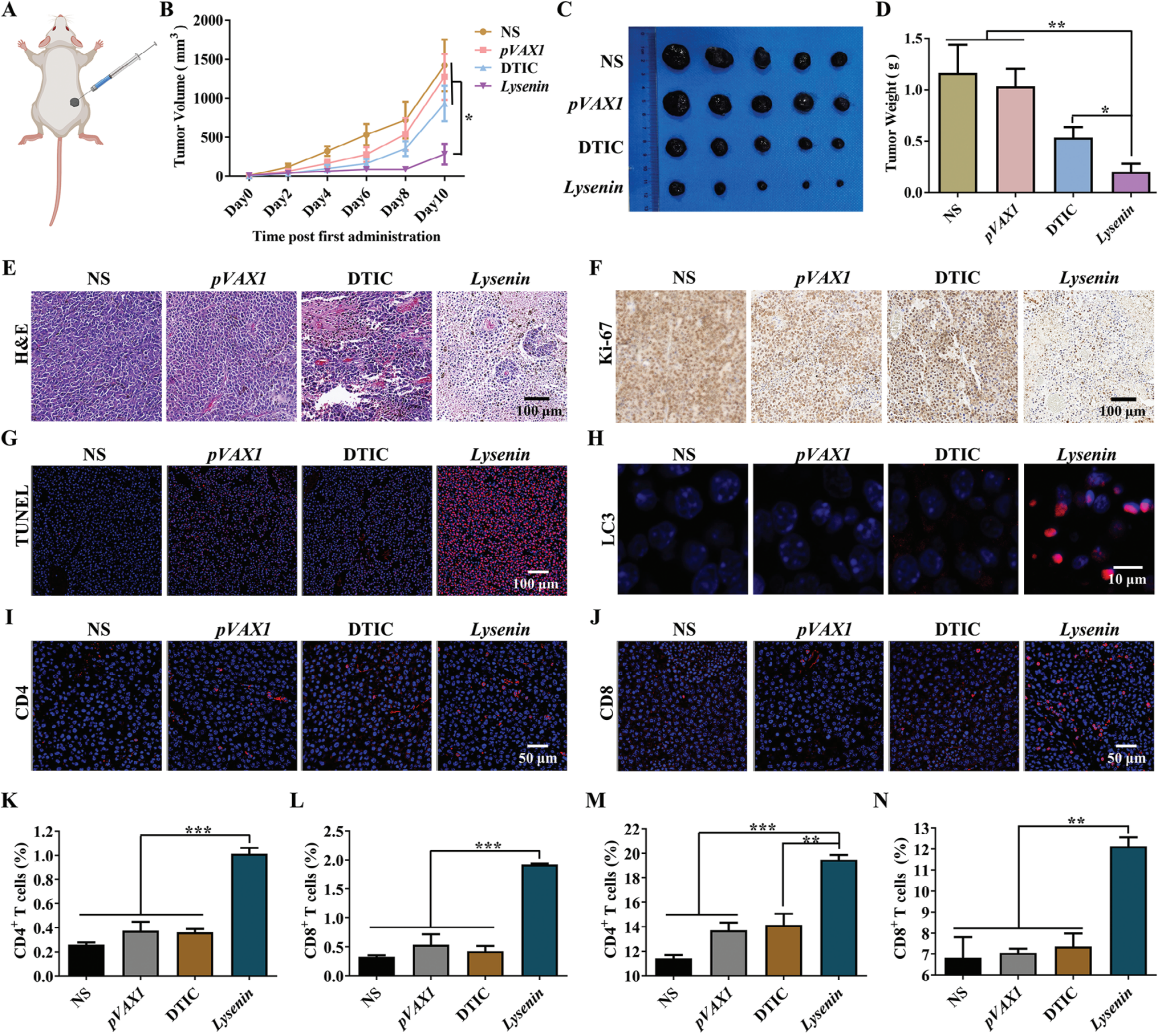
*Lysenin* gene formulation inhibits B16‐F10 melanoma growth in vivo. A) B16‐F10 melanoma subcutaneous tumor model. B) The tumor volumes of each mice group (*n* = 5). ^*^ *p* < 0.05. C) The tumor tissues of each mice group after the treatment (*n* = 5). D) The tumor weights of each mice group after the treatment (*n* = 5). ^*^ *p* < 0.05, ^**^ *p* < 0.01. E) The H&E staining of the tumor tissues. Scale bar, 100 µm. F) The Ki‐67 immunohistochemical staining of the tumor tissues. Scale bar, 100 µm. G) The TUNEL apoptosis detection of the tumor tissues. Scale bar, 100 µm. H) The LC3 immunofluorescent staining of the tumor tissues. Scale bar, 10 µm. I,J) Immunohistochemical fluorescence experiments of CD4 and CD8 of the paraffin sections from the tumor tissues. Scale bar, 50 µm. K,L) Flow cytometry analyses of CD4^+^ and CD8^+^ T lymphocytes in tumors (*n* = 3). ^***^ *p* < 0.001. M,N) Flow cytometry analyses of CD4^+^ and CD8^+^ T lymphocytes in spleens (*n* = 3). ^**^ *p* < 0.01, ^***^ *p* < 0.001.

The in vivo antitumor effect of the *Lysenin* gene formulation was evaluated using B16‐F10 subcutaneous tumor model (**Figure** **3**A). The mice were sacrificed after the tumor volumes reached about 1500 mm^3^. The tumor volumes and weights of the mice in the *Lysenin* group were significantly reduced after the treatment (Figure 3B–D). Hematoxylin and eosin (H&E) staining of the tumor tissues showed a large amount of cell death in the tumor tissues from the *Lysenin* group (Figure 3E). Immunohistochemical staining of the proliferating cell‐associated antigen Ki‐67 demonstrated that *Lysenin* gene formulation attenuated the proliferation ability of B16‐F10 melanoma. (Figure 3F). The nuclear DNA damage was characterized by TUNEL (TdT mediated dUTP Nick End Labeling) apoptosis detection (Figure 3G), and the autophagosome formation was observed by LC3 immunofluorescence staining (Figure 3H), indicating that the formulation could induce cell death in tumor tissues. Therefore, *Lysen*in gene formulation could effectively inhibit the growth of B16‐F10 melanoma in vivo.

Subsequently, we tested the antitumor immune response generated by *Lysenin* gene formulation. CD4^+^ and CD8^+^ T cells, the significant immune cells of the immune system, were often used to characterize effector T cells. We examined the CD4^+^ and CD8^+^ T lymphocytes of the tumor tissues by immunohistochemical fluorescence experiments of the paraffin sections. The results indicated that the CD4^+^ and CD8^+^ T lymphocytes increased in the tumor tissues from the *Lysenin* group (Figure 3I,J). The flow cytometry analyses demonstrated that the proportions of CD4^+^ and CD8^+^ T lymphocytes in the tumors of the *Lysenin* group increased by 2.9 and 4.9‐fold compared with those of the NS group, respectively (Figure 3K,L). Moreover, we examined the activation of the splenic lymphocytes. Flow cytometry analyses of the murine splenocytes showed that the proportions of both CD4^+^ and CD8^+^ T lymphocytes were significantly increased in the *Lysenin* group after treatment compared with those in the NS group (Figure 3M,N), indicating that *Lysenin* gene formulation stimulated the activation of the T lymphocytes in the spleens. Our findings suggested that *Lysenin* gene formulation could activate the antitumor immune response.

**Figure 4:**
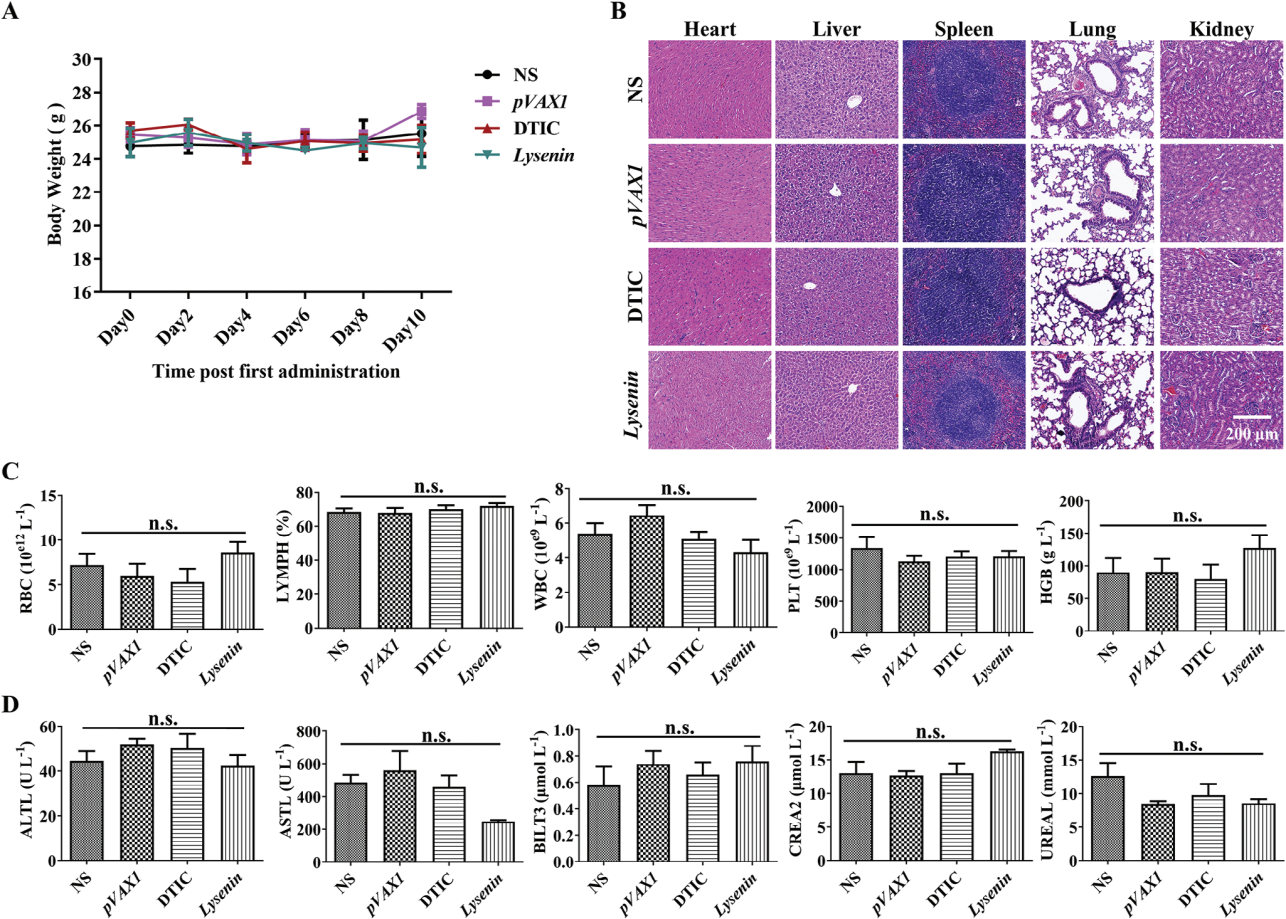
The biosafety evaluation of *Lysenin* gene formulation in mice. A) The statistical data of the murine body weights (*n* = 5). B) H&E staining of the murine vital organs. Scale bar, 200 µm. C)The complete blood count of the mice (*n* = 3). n.s. *p* > 0.05. D)The biochemical blood analysis of the mice (*n* = 3). All data are processed using GraphPad Prism 8. n.s. *p* > 0.05.

Additionally, we studied the biosafety of *Lysenin* gene formulation in mice. The weight data of the mice indicated that the administration of *Lysenin* gene formulation did not affect their growth (**Figure** **4**A). The H&E staining results showed no obvious lesions in the vital organs of the mice (Figure 4B). The complete blood count and biochemical blood analysis of each mice group were within the normal ranges (Figure 4C,D). No obvious adverse effect was observed when the gene formulation was administered intratumorally at a dose of 5 µg plasmids per mouse. To summarize, our data demonstrated that *Lysenin* gene formulation could shield the non‐specific killing of Lysenin on the vertebrate cells, enlightening the design of PFPs‐based therapeutics.

### 2.4 The Suppression of *Lysenin* Gene Formulation on Distant Tumor

**Figure 5:**
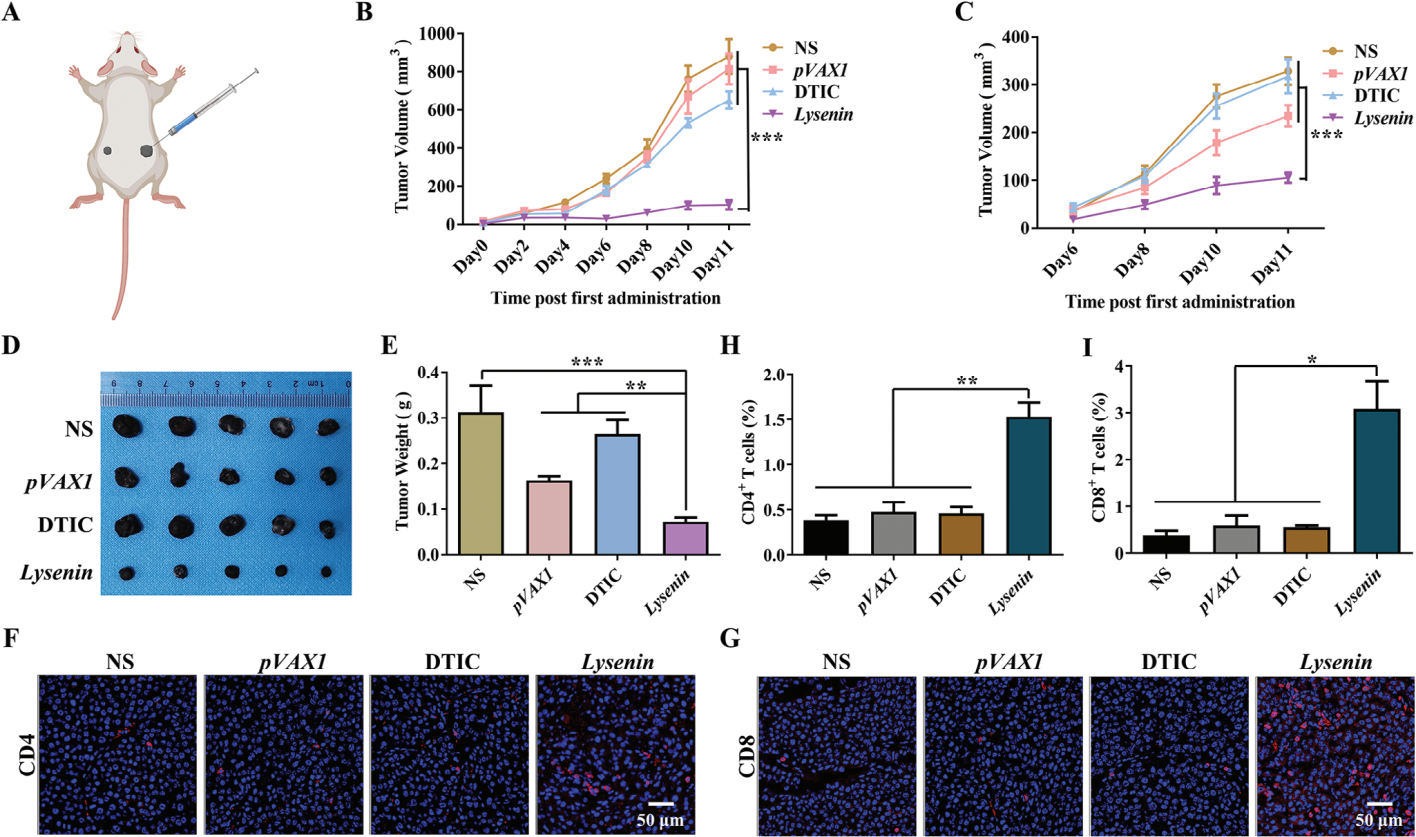
*Lysenin* gene formulation suppresses distant tumor growth. A) The administration of *Lysenin* gene formulation. B) The tumor volumes of the primary tumors (*n* = 5). ^***^ *p* < 0.001. C) The tumor volumes of the contralateral tumors (*n* = 5). ^***^ *p* < 0.001. D) The tumor tissues of the contralateral tumors (*n* = 5), and E) The statistical data of the tumor weights (*n* = 5) by GraphPad Prism8. ^**^ *p* < 0.01, ^***^ *p* < 0.001. F,G) Immunohistochemical fluorescence experiments of CD4 and CD8 of the paraffin sections from the contralateral tumors. Scale bar, 50 µm. H,I) Flow cytometry analyses of CD4^+^ and CD8^+^ T lymphocytes in the contralateral tumors (*n* = 3). ^*^ *p* < 0.05, ^**^ *p* < 0.01.

The results above showed that *Lysenin* gene formulation could induce immunogenic cell death of B16‐F10 murine melanoma cells and activate the antitumor immune response in mice. In order to explore the therapeutic effect of *Lysenin* gene formulation on distant tumors, we inoculated B16‐F10 melanoma tumors on the contralateral side to simulate the formation of metastases 4 d after inoculating the primary tumors (**Figure** **5**A). The tumor volume data of the primary tumors exhibited that this *Lysenin* gene formulation could still effectively inhibit the growth of primary tumors (Figure 5B). During the treatment, we found that the growth rate of the contralateral tumors in the *Lysenin* group was slower than that in other groups (Figure 5C). After the treatment, the contralateral tumors dissected from the mice in the *Lysenin* group were significantly smaller, and the tumor weights were reduced compared with those in other groups (Figure 5D,E). Based on the observed alterations in tumor volumes and weights, it could be preliminarily inferred that the *Lysenin* gene formulation can inhibit the growth of distant tumors.

In addition, we examined the immune responses in the contralateral tumors. The immunofluorescence staining of the paraffin sections from contralateral tumors indicated enhanced infiltration of CD4^+^ and CD8^+^ T lymphocytes within the tumors of the *Lysenin* group (Figure 5F,G). Flow cytometry analyses showed that the proportions of CD4^+^ and CD8^+^ T lymphocytes in the tumors of the *Lysenin* group were enhanced by 3.0 and 7.1‐fold, respectively, compared with those in the NS group (Figure 5H,I).

## 3 Discussion

In this study, we designed a Lysenin‐based gene therapy for cancer treatment. Lysenin, as a defensive factor within earthworm *Eisenia foetida*, has a variety of biological activities.^[^ ^1^ ^]^ Some of its applications have been explored, primarily focusing on the following aspects: 1) Based on its binding properties to SM, Lysenin has been employed to visualize the distribution and dynamics of SM in cells to investigate the function and metabolism of SM, which is of great significance for studying the occurrence and development of various diseases.^[^ ^36^, ^37^, ^38^, ^39^, ^40^, ^41^, ^42^ ^]^ 2) According to its pore characteristics, Lysenin is used as an analytical tool for studying substances. For example, it is used to study the interaction of polyvalent inorganic and organic cations, random sensing of polypeptides and DNA molecules, molecular translocation, and as a sensor of ions and molecules.^[^ ^9^, ^43^ ^]^ Our research focused on the antitumor activity of Lysenin and explored its potential in cancer therapy, which would expand its application in cancer treatment.

PFPs have many biological functions. However, due to their cytolysis to normal cells and possible immunotoxicity, direct administration of these proteins can cause tissue necrosis, hemolysis, immunotoxicity, and other side effects, which hinder their application in cancer therapy.^[^ ^44^ ^]^ Currently, there is some research to address the toxicity by enhancing the targeting or efficacy via modifying PFPs, such as fusing antibody, receptor‐binding domain, or protease‐recognition domain.^[^ ^45^, ^46^, ^47^ ^]^ Nevertheless, the toxicity problem still needs to be solved. In this study, we employed gene therapy based on Lysenin to treat tumors, which could effectively inhibit tumors without obvious adverse effects. Because the gene formulation had a different material foundation from therapeutic proteins, this gene formulation only possessed lysis ability once the gene was expressed in the tumor cells. Recently, some attempts have been made to address the PFPs‐associated toxicity using gene therapy, which could also support our study.^[^ ^48^, ^49^ ^]^ Our gene therapy approach provides a reliable solution to mitigate the PFPs‐associated toxicity of Lysenin, which would promote the development of novel therapeutics based on Lysenin and other PFPs.

Meanwhile, we found that the secretory signal peptide could influence the intracellular distribution and function of Lysenin in our study. The exogenous PFPs usually acted on cell plasma membrane. The signal peptide altered the distribution and provided a new acting site of Lysenin. In addition, the signal peptide‐mediated Lysenin had strong antitumor capacity by directly killing tumor cells and activating an antitumor immune response, indicating that it had different antitumor mechanisms. Our results demonstrated that signal peptide played a significant role in the gene therapy based on PFPs. The design of signal peptide‐modified Lysenin would inspire the novel drug design and enlighten the development of PFPs‐based cancer treatment.

Moreover, we employed biodegradable polymeric nanoparticles to mediate the *Lysenin* gene therapy. By using the non‐viral vector, the delivered genes did not integrate into the genome, which would reduce potential bio‐risk. In addition, the polymeric nanoparticles were biodegradable, which would not accumulate in the body and cause toxicity. The administration of polymeric nanoparticle‐mediated *Lysenin* gene formulation could effectively inhibit tumor growth and did not induce any obvious adverse effect, exhibiting that the designed *Lysenin* gene formulation had potential clinical application.

## 4 Conclusion 

In this study, we designed a non‐viral gene formulation based on Lysenin derived from earthworm *Eisenia foetida* for cancer therapy. Our gene formulation could efficiently destroy the tumor by directly killing cancer cells and activating antitumor immunity without obvious adverse effects. Our work demonstrated a design strategy for nanoparticle‐mediated nanomedicine with potential clinical application in cancer therapy, which would lead to the future development of therapeutics based on PFPs.

## 5 Experimental Methods

### Plasmid Construction and Identification

The protein sequence of Lysenin was derived from the database National Center for Biotechnology Information (GenBank: BAA21518.1) and was codon‐optimized according to the mammalian expression system. To construct the recombinant *Lysenin^SP−^* plasmid, its signal peptide was removed. In *Lysenin* plasmid, its signal peptide was replaced by that of IL‐2 (IL‐2 signal peptide sequence: ATGTACAGGATGCAACTCCTGTCTTGCATTGCACTAAGTCTTGCACTTGTCACGAATTCG). To facilitate the detection of protein expression, a flag sequence was added after the *Lysenin* open reading frame. The nucleic acid sequence was synthesized and ligated into the mammalian transient expression vector *pVAX1*. The *Lysenin^SP−^‐GFP* and *Lysenin‐GFP* plasmids were constructed based on *Lysenin^SP‐^* and *Lysenin* plasmids, respectively. All the constructed recombinant plasmids were identified by DNA sequencing.

### Preparation of Gene Formulations

The polymeric gene delivery system (DPP nanoparticle) was self‐assembled from mPEG‐PDLLA and DOTAP. The synthesis method was referred to the previous article.^[^ ^28^ ^]^ Briefly, 18 mg mPEG‐PDLLA and 2 mg DOTAP were dissolved in dichloromethane (DCM), respectively. Then, they were mixed, and the lipid film was formed by removing the DCM via rotary evaporation. The DPP nanoparticles were obtained by rehydrating the film with double‐distilled water and were adjusted to the final concentration of 2 mg mL^−1^ for further use.

### Characterization of Gene Formulations

To prepare the gene formulations, the DPP nanoparticles and plasmids were mixed with different mass ratios and incubated at room temperature for 30 min. After the incubation, the nanocomplexes were electrophoresed on 1% (w/v) agarose gel and detected using ChemiDoc Imagers (Bio‐RAD ChemiDoc XRS, USA). The hydrodynamic size and zeta potential of the gene formulations were analyzed by dynamic light scattering using a Zetasizer Nano ZS (Malvern Instruments, Worcestershire, UK). The morphology was observed via TEM(HT7800, Hitachi, Japan), and the transfection ability on B16‐F10 melanoma cells was evaluated through the reporter gene *GFP*.

### Cell Transfection

The B16‐F10/A375 melanoma cells were obtained from the American Type Culture Collection (ATCC; Manassas, VA, USA). Cells were cultured in Dulbecco's Modified Eagle Medium (DMEM) supplemented with 10% (v/v) fetal bovine serum (FBS; Gibco, Grand Island, CA, USA) under 5% CO_2_ at 37 °C. Cell transfection was mediated by DPP polymeric nanoparticles. The cell medium was changed to the serum‐free DMEM medium. DPP nanoparticles and plasmids were diluted with the serum‐free DMEM medium, mixed, and incubated at room temperature for 30 min. After the incubation, the mixture was added to the cell cultures, mixed gently, and incubated at 37 °C, 5% CO_2_, for 7 h. Then, the medium was changed to DMEM medium containing 10% FBS.

### Detection of Cell Viability

B16‐F10 cells were seeded in 24‐well plates with 6 × 10^4^ per well and cultured for 16 h. Then, the gene formulation was added to the cell cultures. After treatment for 24 h, the cells were stained with Calcein AM according to the instruction (Beyotime, Shanghai, China) and observed with a fluorescent microscope. The cell density was analyzed by Image J and GraphPad Prism 8.

### LDH Release Assay

The cells were cultured in 48‐well plates and treated by gene formulations for 24 h. LDH release assay was performed with the CytoTox 96 Non‐Radioactive Cytotoxicity Assay (Promega, Madison, USA), and the whole process was protected from light. Initially, 20 µL of 10 × Lysis Solution was added to the positive control wells and incubated for 45 min to generate a Maximum LDH Release Control. Next, 50 µL aliquots from all tests and control wells were transferred to a fresh 96‐well flat clear bottom plate, and 50 µL of the CytoTox 96 Reagent was added to each sample aliquot, mixed well, and incubated at room temperature for 30 min protected from light. Then, 50 µL of Stop Solution was added to each well. Large bubbles were popped using a syringe needle, and the absorbances at 490 nm were recorded. Percent cytotoxicity = Experimental LDH Release (OD_490_)/Maximum LDH Release (OD_490_) × 100%.

### Western Blotting (WB) Analyses

The cells were harvested and lysed by RIPA lysis buffer (Biosharp, Anhui, China) containing protease inhibitors (MCE, State of New Jersey, USA) and phosphatase inhibitors (EpiZyme, Shanghai, China). The protein concentration was determined using a BCA protein assay kit (Biosharp, Anhui, China). A 10–30 µg aliquot of the total proteins from each sample was taken, separated by 15% sodium dodecyl sulfate‐polyacrylamide gel electrophoresis, and transferred to polyvinylidene fluoride membranes. The membrane was blocked with 5% non‐fat milk or BSA at room temperature for 2 h, probed with primary antibodies (anti‐Flag, anti‐LC3, anti‐pMLKL, anti‐pRIP3: Abcam, Cambridge, England; anti‐β‐tubulin, anti‐β‐actin: Abmart, Shanghai, China), and incubated with the corresponding secondary antibodies (Abmart, Shanghai, China). The immunoblot proteins were detected by enhanced chemiluminescence (Biosharp, Anhui, China). The assays were repeated three times, and the gray values were analyzed by Touch Imager and GraphPad Prism 8.

### Immunofluorescence Staining

Cells were fixed with paraformaldehyde at room temperature for 10 min, washed with phosphate‐buffered saline (PBS) for 5 min each time, and blocked with the serum of the secondary antibody‐derived host (ZSGB‐BIO, Beijing, China) at room temperature for 1 h. After blocking, cells were incubated with the primary antibody overnight at 4 °C. Immuno‐response products were labeled with fluorescent secondary antibodies (Abcam, Cambridge, England) and observed under a fluorescence microscope.

### Extraction and Cultivation of Murine Bone Marrow‐Derived Dendritic Cells (BMDCs)

The C57BL/6J mouse was killed by cervical dislocation. The femur and tibia were taken and soaked in 75% ethanol for 2–3 min. Then, they were washed twice with PBS solution and soaked in RPMI‐1640 medium. After removing the leg musculature, the femur and tibia were cut off. The bone marrows were flushed into the medium and dispersed using the syringe. The mixtures were filtered by a 70 µm cell sieve, and the red blood cells were removed using the Red Blood Cell Lysis Buffer (Biosharp, Anhui, China). The obtained bone marrow cells were resuspended in RPMI‐1640 medium (containing 10% FBS, 25 ng mL^−1^ rmGM‐CSF, and 10 ng mL^−1^ rmIL^−4^) and seeded into a 12‐well cell cultivation plate for incubation at 37 °C under 5% CO_2_ condition for 3 d. Half of the cultivation medium was removed on the third day, followed by adding a fresh RPMI‐1640 medium containing 10% FBS, rmGM‐CSF, and rmIL‐4. On the fifth day, the BMDCs were harvested and co‐cultured with B16‐F10 cells that were treated with *Lysenin* gene formulation.

### Transmission Electron Microscopy (TEM) Imaging for Cell Samples

The cells were digested with 0.25% trypsin and collected by centrifugation. After the supernatant was discarded, the cell pellet was added with 2.5% glutaraldehyde fixative and fixed at room temperature for 5 min. The cell suspension was transferred to a 1.5 mL centrifuge tube and centrifuged at 2000 rpm for 10 min. The supernatant was discarded carefully with a pipette. The cells were washed with PBS twice. Then, the cell pellets were resuspended in GT2 solution, pre‐warmed to 40 °C, and centrifuged at 3000 rpm for 10 min. The supernatant was carefully discarded with a pipette as soon as possible. After that, the cap of the tube was sealed, and the tube was transferred to 4 °C for 10 min. The glutaraldehyde fixative was slowly added to the pellet along the tube wall. Subsequent TEM sample preparation was assisted by the Electron Microscopy Platform, School of Basic Medicine and Forensic Medicine, Sichuan University.

### Mouse Models

C57BL/6J mice (female, 6 weeks old) were purchased from the Beijing HFK Bio‐Technology Laboratory Animal Center, China. All animal experiments were approved by the Animal Ethics Committee of Sichuan University (Approval No. 20230112005). After 5 d of adaptive feeding, the B16‐F10 melanoma cells were subcutaneously inoculated on the right side of the back of the mice with an inoculation amount of 3 × 10^5^ cells per mouse. After 7 d of tumor inoculation, the drugs were administrated intratumorally once every other day (*n* = 5). The mice in the control group were injected with 100 µL PBS per mouse. The mice in the positive group were injected with 120 µg DTIC (Bidepharm, Shanghai, China) per mouse. The mice in the *pVAX1* and *Lysenin* groups were administrated DPP nanoparticles containing 5 µg plasmids per mouse. Tumor diameters were measured by a caliper. The tumor volume was calculated as large diameter × small diameter^2^ × 0.52. When the tumor volume approached ≈1500 mm^3^, the mice were euthanized. For contralateral B16‐F10 metastatic melanoma tumor experiments, 6 × 10^5^ melanoma cells per mouse were inoculated on the left back to simulate the metastatic tumor on the fourth day of the primary tumor inoculation. Metastases on the left side were not administered throughout the treatment.

### The Complete Blood Count and Biochemical Blood Analysis

At the end of the treatment, the blood was collected from the abdominal aorta and divided into two copies. One copy was used for the complete blood count, and the other was used for the biochemical blood analysis. The samples for complete blood count were collected using anticoagulant tubes containing EDTAK2 (KANGJIAN, Jiangsu, China). The samples for biochemical blood analysis were collected using separating gel‐procoagulant tubes (KANGJIAN, Jiangsu, China) and centrifuged at 1800 *g* for 10 min. The upper serums were transferred to 1.5 mL centrifuge tubes for follow‐up tests. The test was entrusted to the Pathology Department of Chengdu Huaxi Haiqi Pharmaceutical Technology Co., Ltd.

### Immunohistochemistry Assays

Animal tissues were stripped, soaked in a 4% paraformaldehyde solution, and fixed for 48 h. The tissue dehydrator was used for dehydration, transparency, and wax dip. Paraffin was used as an embedding agent, and the embedded tissues were cut into slices (5 µm thickness) by a paraffin microtome. After immersing the slices twice in xylene for 15 min each, soaking them in gradient ethanol for 10 min, respectively. H&E staining was performed with hematoxylin for 5 min and eosin for 40 seconds. The differentiation and bluing were employed with 1% hydrochloric alcohol and 1/400 ammonia, respectively. Disodium citrate was used as a repair solution, and a 3% hydrogen peroxide solution was used to block the endogenous peroxidase. The normal serum from goat (ZSGB‐BIO, Beijing, China) is used as the blocking and diluent buffers. The slides were blocked at room temperature for 1 h and incubated with diluted primary antibodies at 4 °C overnight. After washing with PBS, the sections were incubated with the corresponding HRP‐conjugated IgG at room temperature for 1 h. The DAB reaction was observed microscopically and terminated by immersing the sections in PBS. The nuclei were labeled with DAPI. TUNEL staining of tumor tissues was carried out according to the manufacturer's instructions (YEASEN, Shanghai, China).

### Flow Cytometry Analyses of the Murine Tissues

After the mice were sacrificed, the spleens and tumors were dissected. The tissues were ground into a cell suspension in PBS and filtered with a 70 µm cell sieve. The erythrocytes were removed by the Red Blood Cell Lysis Buffer (Biosharp, Anhui, China). The tissue cells were washed three times with PBS and counted by cell counting apparatus. 1 × 10^6^ cells from each sample were stained with PE anti‐Mouse CD4 (BioLegend, San Diego, CA, USA) and APC anti‐Mouse CD8 (BioLegend, San Diego, CA, USA). After staining, analyses were performed by flow cytometry.

### Statistical Analysis

All statistical analyses were performed using GraphPad Prism 8. Data analyses employed unpaired *t*‐tests (and nonparametric tests) with two‐tailed *P* values and one‐way ANOVA (and nonparametric or mixed). The significance thresholds were considered significant (^*^ *p *< 0.05), highly significant (^**^ *p* < 0.01), or extremely significant (^***^ *p* < 0.001).

## Conflict of Interest

The authors declare no conflict of interest.

## Author Contributions

M.R. and L.Y. contributed equally to this work. M.G. designed, supervised the project, and revised the manuscript. M.R and L.Y. analyzed the data and drafted the manuscript. M.R., L.Y., L.H, J.W, W.Z, C.Y., S.Y. and H. C. performed the experiments. M.H. visualized and revised the manuscript.

## Data Availability

The data that support the findings of this study are available from the corresponding author upon reasonable request.
